# Investigation of Chemical Compounds and Evaluation of Toxicity, Antibacterial, and Anti-Inflammatory Activities of Three Selected Essential Oils and Their Mixtures with Moroccan Thyme Honey

**DOI:** 10.3390/foods11193141

**Published:** 2022-10-09

**Authors:** Mouna Mekkaoui, El Houcine Bouidida, Hanae Naceiri Mrabti, Ahmed Ouaamr, Learn-Han Lee, Abdelhakim Bouyahya, Yahya Cherrah, Katim Alaoui

**Affiliations:** 1 Pharmacodynamics Research Team ERP, Laboratory of Pharmacology and Toxicology, Faculty of Medicine and Pharmacy, University Mohammed V, Rabat 554, Morocco; 2 National Laboratory of Drugs Controlled, Agdal, Rabat BP 623, Morocco; 3 Laboratory of Pharmacology and Toxicology, Bio Pharmaceutical and Toxicological Analysis Research Team, Faculty of Medicine and Pharmacy, University Mohammed V, Rabat 554, Morocco; 4 High Institute of Nursing Professions and Health Techniques, ISPITS, Tiznit 85000, Morocco; 5 Novel Bacteria and Drug Discovery Research Group (NBDD), Microbiome and Bioresource Research Strength (MBRS), Jeffrey Cheah School of Medicine and Health Sciences, Monash University Malaysia, Subang Jaya 47500, Selangor, Malaysia; 6 Laboratory of Human Pathologies Biology, Department of Biology, Faculty of Sciences, University Mohammed V, Rabat 554, Morocco

**Keywords:** honey, essential oils, synergy, antibacterial activity, anti-inflammatory activity

## Abstract

Throughout history, honey has been used to treat various diseases. The present work examined and assessed the in vivo anti-inflammatory potential of Moroccan thyme honey and its association with essential oils from three selected plants: *Origanum vulgare* L.; *Mentha spicata* L.; *Eucalyptus globulus* L. The chemical composition of the essential oils was studied, and preliminary toxicity, in vitro anti-inflammatory, and antibacterial tests were conducted. Then the anti-inflammatory effect was determined by applying carrageenan and an experimental trauma-induced paw edema test in rats. The essential oils were rich in phytochemicals and showed significant antibacterial activity against four selected ATCC bacterial strains. The results revealed the significant anti-inflammatory potential of honey and mixtures with essential oils and indicated higher efficiency of mixtures compared to honey alone. It can be concluded that the mixtures of honey and essential oils have advantageous anti-inflammatory effects and may be used for treating different types of inflammation in humans after certain clinical trials.

## 1. Introduction

Inflammation is the natural response of the immune system to pathogens, during which various cellular and humoral immune defenses develop and biological changes occur, such as increased blood flow and capillary distillation, leukocyte infiltration, and the release of localized chemoattractants to recruit immune cells. The primary goals of these changes are to eliminate pathogens and repair damaged tissue [1,2]. However, unresolved inflammation resulting from chronic bacterial infection, aging, or obesity paves the onset of low-grade chronic inflammation, which subsequently develops various chronic diseases [3]. In this regard, supporting the use of natural products to resolve inflammation is the main reason for investigating these products.

Honey is known for its richness in bioactive molecules and essential minerals, which have several healing properties [4]. Many trials have been conducted to investigate the healing effect of honey, and it has been proven that honey exhibits antimicrobial, wound healing, anti-inflammatory, and antioxidant activities, as well as boosting the immune system [4,5,6,7,8,9]. The anti-inflammatory and wound healing properties are known to be the vital bioactivities of honey, and most types of honey have been proven to have anti-inflammatory properties in both in vitro and in vivo tests [10]. Honey’s anti-inflammatory action and stimulatory effects on granulation and epithelialization help rapidly reduce pain and edema [11,12]. In fact, some compounds like prostaglandins and nitric oxide are major players in the inflammation process, and honey is known to increase nitric oxide end products and decrease the prostaglandin levels [13,14]. Furthermore, protease activity; as a result of extra inflammatory reactions, can either slow or stop healing by destroying growth factors, protein fibers, and fibronectin in wounds. The anti-inflammatory activity of honey can eliminate this obstacle because of non-neutral pH [15].

In Morocco, recent studies have revealed the various uses of Moroccan honey in biological applications. El-Haskoury et al. studied carob honey for its antioxidant properties [16]. Imtara et al. covered the antioxidant and wound-healing effects of Tulkarm honey and *Thymus vulgaris* honey [17,18]. The study by Elamine et al. reviewed the physicochemical characteristics and antioxidant activities of Moroccan Zantaz honey [19]. El-Guendouz et al. evaluated *Capparis spinosa* honey and propolis for their antioxidant and diuretic activities [20]. Botoub et al. researched the antioxidant and enzyme inhibitory of two types of *Euphorbia* honey [21]. However, only a few studies covered the anti-inflammatory activity of Moroccan honey. Plants also are used for their anti-inflammatory properties by many traditional practitioners across the world, and numerous studies highlighted the effectiveness of their essential oils in reducing inflammation. Morocco is known for its rich and varied flora, with more than 4,500 species and 600 plants possessing medicinal and aromatic properties [22,23]. Thus, many recent studies have investigated the pharmacological properties, such as antimicrobial [24,25,26], anticancer [27,28], antidiabetic [29,30,31], and analgesic [32,33] effects of various Moroccan plants, and several studied their anti-inflammatory potential [34,35,36]. Rare are the studies carried out on the association between honey and essential oils (EOs) or plant extracts [18,37,38,39,40,41]. None of them investigated the synergistic effect between honey and EOs for the treatment of inflammation. Therefore, the main aim of the present work is to evaluate the anti-inflammatory effects of oregano, spearmint, and eucalyptus essential oils mixed with Moroccan thyme honey on two types of edemas induced in Wistar rats. Antibacterial potential and toxicity (acute and subacute) were also evaluated, and the chemical composition of essential oils was quantified and discussed. This work presents and discusses the first study regarding the in vivo anti-inflammatory potential of mixtures of honey and EOs.

## 2. Materials and Methods

### 2.1. Honey Sample Collection

Thyme honey samples were obtained from local beekeepers in Ida Ou Kazzou (30°54′39″ N 9°36′7″ W), a small town in Tamanar, a rural municipality in Essaouira province. All processing steps, from harvesting to packaging, were carried out using traditional methods. The samples were stored in sealed jars, labeled and dated, then kept at room temperature ±29 °C until the end of the analysis.

The studies were carried out before exceeding 3 months from the collection date. Multiple methods were applied to estimate the collected honey samples’ physicochemical properties; all were performed as described in a previous study [37].

### 2.2. Essential Oils Selection and Their Chemical Composition

Essential oils used in this study were selected based on bibliographical research in Databases such as Scopus, PubMed, and ScienceDirect. The search was carried out with “essential oils” and “anti-inflammatory” as the main keywords. From this search, only results, including an animal model, were considered. In a second step, “honey mixture” or “honey and essential oil” were added to the same previous keywords: Works devoted to the association of EO and honey for anti-inflammatory purposes have been selected. Along with the bibliographical research, a survey was conducted in five Moroccan cities aimed at beekeepers, herbalists, and traditional healers. Plants species and honey types used in Morocco for their beneficial healing effect for wound treatment were reported in a previous study [37]. Three EOs with high anti-inflammatory potential were selected for this study.

Essential oils of three plants, *Eucalyptus globulus* (*E. globulus*), *Mentha spicata* (*M. spicata*), and *Origanum vulgare* (*O. Vulgare*), were provided by EDEPAM, a company located in the city of Kenitra, which is specialized in the identification, distillation, and exploitation of aromatic and medicinal plants.

The chemical components of the EOs were determined by gas chromatography-mass spectrometry (GC-MS) analysis following the method described in a previous study [37]. Briefly, a Hewlett-Packard (HP6890) GC instrument (Santa Clara, CA, USA) coupled with an HP5973 MS and equipped with a 5% phenylmethyl silicone HP-5MS capillary column (30 m × 0.25 mm × film thickness of 0.25 μm) was used in GC analysis. The used column temperature increased from 50 °C for 5 min to 200 °C with a 4 °C/min rate. Helium with a 1.5 mL/min flow rate and a split mode (flow: 112 mL/min, ratio: 1/74.7) was the used carrier gas. The hold time was 48 min, and the injector and detector were both 250 °C. The machine was led by a computer system of the “HP ChemStation” type, managing the functioning of the machine and allowing us to follow the evolution of chromatographic analyses. Diluted samples (1/20 in methanol) of 1 μL were injected manually. In addition, 70 eV ionization voltage, 230 °C ion source temperature, and a 35–450 (m/*z*) scanning range were the MS operating conditions. Finally, the identification of different compounds was carried out by comparing MS spectra with the library and matching the Kovats index (Library of NIST/EPA/NIH MASS SPECTRAL LIBRARY Version 2.0, 1 July 2002). The quantification of the different compounds was obtained by internal normalization of the total area of peaks detected in each chromatogram.

### 2.3. Antibacterial Activity

#### 2.3.1. Preparation of Bacterial Strains

Bacterial species examined are *Escherichia coli* (ATCC 25922), *Staphylococcus aureus* (ATCC 29213), *Salmonella Typhimurium* (ATCC 700408), and *Listeria monocytogenes* (ATCC 13932). The bacterial strain method of preparation was reported in previous work. Briefly, a loopful from the frozen stock (−20 °C) was inoculated in Mueller-Hinton Agar and then incubated at 37 °C for 24 h [37].

#### 2.3.2. Disc Diffusion Assay

Preliminary evaluation for the antibacterial potential of the studied EOs was carried out by disc diffusion assay following the previously published protocol. Briefly, the culture suspension was inoculated by swabbing on optimal culture media (Mueller-Hinton Agar), and EO (mixed with 5% of DMSO) was deposited on each plate. Chloramphenicol (30 µg) was used as a positive control, while DMSO (10 µL; 5%) was used as a negative control.

The plates were incubated at the following growth conditions: 37 °C for 24 h. After incubation, the inhibitory diameters were measured in millimeters, and the results were expressed as means ± standard deviation of three replicates [42].

#### 2.3.3. Determination of MIC and MBC

The minimum inhibitory concentration (MIC) was performed by microbroth dilution in 96 well-microplates, as described in a previous study [37]. Briefly, decreasing concentrations of each EO were prepared in DMSO using the serial two-fold dilution method in each microplate row. Then, 20 μL of 0.5 MacFarland bacterial suspensions and 160 μL of Mueller-Hinton broth (MHB, Biokar, Beauvais, France) were added, respectively, and the microplates were incubated at 37 °C for 24 h. Afterward, the bacterial growth was checked by adding 40 μL of 2, 3, 5- triphenyl tetrazolium chloride (TTC) (Sigma-Aldrich, Switzerland) with a concentration of 0.2 g/mL, followed by incubation for 30 min at 37 °C. The TTC stains the bacteria red, indicating the wells showing bacterial growth [43]. The microplate wells containing the lower concentration of EO and didn’t show visible bacterial growth were considered the MIC. However, the determination of minimum bactericide concentration (MBC) was performed by sub-culturing 50 μL from the microplate well that didn’t present bacterial growth on Mueller-Hinton Agar (Biokar, Beauvais, France), then the plates were incubated at 37 °C for 24 h. The lower concentration that does not present growth in media was considered the MBC [37]. In this study, chloramphenicol (30 μg/disc) (Sigma-Aldrich) was used as a reference test.

### 2.4. Acute and Subacute Toxicity Studies of the Essential Oils

#### 2.4.1. Animals

The toxicity study was carried out using 60 male and female Swiss mice (20–25 g, aged 4–5 months) for the acute toxicity test and 16 male and female Wistar rats (230–250 g, aged 3–5 months) for the subacute oral toxicity test. The animals were kept at constant room temperature and were fed ad libitum with standard feed and water during the study (14 days for the acute toxicity test and 28 days for the subacute test). The animals were obtained from the animal experimental center of Mohammed V- Souissi University, Medicine and Pharmacy Faculty Rabat. All the experiments were conducted between 9.00 a.m. and 4 p.m. with normal room light and temperature (22 ± 1 °C). The studies were carried out in accordance with the guidelines set by the National Academy of Sciences “Guide for the care and Use of Laboratory Animals”, published by the National Institutes of Health, and in accordance with the guidelines of the Declaration of Helsinki. The study was approved by the Ethics Committee from Mohammed V University (Protocol code # UA-2021-02, Date of approval 26 May 2021).

#### 2.4.2. Acute Toxicity

Median lethal dose (LD_50_) values were determined according to the method described by Litchfield and Wilcoxon [44]. Ten groups of mice of both sexes (n = 6; 3 males and 3 females) were administered or not intraperitoneally (i.p.) single dose at different concentrations (500; 1000; 1500; 2000; 3000; and 5000 mg/kg; i.p.), for essential oil from *M. spicata*, (80; 150; 250; 350; 450; and 500 mg/kg, i.p.), for essential oil from *O. vulgare*, and (1500; 1650; 1750; 1850; 1950; and 2000 mg/kg; i.p.), for essential oil of *E. globulus*. The control group received only distilled water. After a single dose, mice were placed in individual clear plastic boxes and observed continuously for 24 h at 6-h intervals for possible side effects and were monitored for 14 days afterward. The percentage of mortality was calculated using a program by Boniface et al., which calculated the percentage of mortality as a function of the dose administered [45]. The number of dead animals during the test period was expressed as a percentile, and LD_50_ was obtained by probit test with percent mortality used as a function of the log doses [46,47].

#### 2.4.3. Subacute Oral Toxicity

According to the OECD Guidelines for testing of Chemicals, subacute toxicity was performed after administrating a high dose of 5000 mg/kg of essential oil of *M. spicata*, 1795.7 mg/kg of essential oil of *E. globulus,* and 2000 mg/kg of essential oil of *O. vulgaris* for 28 days.

Rats were divided into four groups (n = 4) to perform the study. The animals in the test groups received a daily dose for 28 days, which was given using a rigid gastric cannula. The control group received the same amount of liquid (i.e., water) by gavage daily. All animals were observed daily during the 28 days of intervention. Body weight was controlled every two days, and toxic signs were monitored daily. The percentage of weight gain was calculated as follows:

$$\mathrm{eight}\;\mathrm{gain}\;\mathrm{percentage}\;\left(\%\right)=\frac{\left(\mathrm{starting}\;\mathrm{weight}-\mathrm{current}\;\mathrm{weight}\right)}{\mathrm{starting}\;\mathrm{weight}}\times 100$$

### 2.5. Anti-Inflammatory Activity

#### 2.5.1. Animals and Groups

The study was performed on 72 adult rats of Wistar strain weighing between 200 and 250 g and aged 3–5 months. These animals were obtained from the central animal facility of the Faculty of Medicine and Pharmacy of Rabat. The temperature of the animal house was maintained at 20 °C with a lighting cycle of 12 h of light/12 h of darkness [48,49].

Rats were divided into 12 groups (n = 6); 6 groups will serve for each anti-inflammatory test model.

For the carrageenan-induced paw edema test:

- Groups I was treated daily for two months with the mixture of thyme honey (TH) and essential oil of *O. vulgare*;
- Group II received, for two months, a daily dose of the mixture of TH and essential oil of *M. spicata*;
- Group III received, for two months, a daily dose of the mixture of TH and essential oil of *E. globulus*;
- Group IV was fed daily for two months with TH;
- Group V served as positive control and received indomethacin (10 mg/Kg) one hour before the induction of edema;
- Group VI served as the negative control.

For experimental trauma-induced hind paw edema test:

- Groups I was treated daily for two months with the mixture of TH and essential oil of *O. vulgare*;
- Group II received, for two months, a daily dose of the mixture of TH and essential oil of *M. spicata*;
- Group III received, for two months, a daily dose of the mixture of TH and essential oil of *E. globulus*;
- Group IV was fed daily for two months with TH;
- Group V served as positive control and received indomethacin (20 mg/Kg) one hour before the induction of edema;
- Group VI served as the negative control.

#### 2.5.2. Pretreatment Protocol and Dosing Regimen for Thyme Honey and Mixtures between the Honey and Essential Oils of *M. spicata, E. globulus, and O. vulgare.*

For the groups pretreated with mixtures (honey-essential oil), preparations were formulated daily according to the results of the acute and subacute oral toxicity tests and following the method described by Assagaf et al. [50].

For the mixture of TH and essential oil of *O. vulgare*, 250 mg/kg of *O. vulgare* EO was combined and homogenized with TH (1:1).

For the mixture of TH and essential oil of *M. spicata*, 2000 mg/kg of *M. spicata* EO was mixed with TH (1:1); and for the mixture of TH and essential of *E. globulus*, 1795.7 mg/kg of *E. globulus* EO was combined with TH (1:1).

For the groups treated with TH, the daily dose administered was 3 g per kg of body weight (g/kgbw). This dose was calculated according to the local human consumption, equivalent to two tablespoons.

The largest and smallest rats were weighed, and the average weight was used to calculate the maximum dosage for all animals in the same experimental group.

Mixtures were prepared daily during the entire duration of the pretreatment (2 months).

Animals were observed daily, and body weight was controlled every week.

#### 2.5.3. In Vivo Anti-Inflammatory Test Models

To evaluate the anti-inflammatory activity, we chose as experimental protocol a preventive inflammation model. The first test is that of carrageenan-induced paw edema, and the second of experimental trauma-induced hind paw edema. Both principles are based on the induction of edema either by injecting carrageenan (a phlogogenic agent) or by experimental trauma, as described in other studies [51,52,53,54].

Carrageenan-Induced Paw Edema Test

The induction of edema is performed in all groups by injecting a volume of 0.05 mL of carrageenan diluted to 1% in a 9% NaCl solution into the plantar aspect of the rat’s left paw.

Volume changes of both legs for each rat were measured using a LE 7500 digital plethysmometer, controlled by SeDaCOM software, just before the carrageenan injection, then at 1 h 30 min, 3 h, and 6 h after edema induction. The right hind paw is considered the control without treatment [51].

The anti-inflammatory activity was expressed as percentage inhibition of edema thickness (% INH) in treated animals versus the control group:

$$\%\;\mathrm{INH}=\frac{(\mathrm{mean}\left(V_{L}-V_{R}\right)\mathrm{control}-\mathrm{mean}\left(V_{L}-V_{R}\right)\mathrm{treated}}{\mathrm{mean}\left(V_{L}-V_{R}\right)\mathrm{control}}\times 100$$

with: V_L_: volume of the left paw. V_R_: volume of the right paw.

Experimental Trauma-Induced Hind Paw Edema

The induction of inflammation was performed by dropping a weight of 50 g onto the dorsum of the left hind paw of all animals [55]. Techniques for assessing edema and calculating the percentage of edema inhibition were performed identically to the previous test.

#### 2.5.4. In Vitro Anti-Inflammatory Activity

In vitro evaluation was performed by 5-Lipoxygenase (5-LOX) inhibition assay.

Lipoxygenase inhibitory activity of thyme honey and essential oils of *M. spicata*, *O. vulgare,* and *E. globulus* was evaluated by following the linoleic acid oxidation at 234 nm, according to the published method by Andrade et al. [56] with some modifications. Briefly, for each essential oil, 20 µL (solubilized in Tween 80) and 20 µL of 5-LOX from Glycine max (100 U/mL) were pre-incubated with 200 µL of phosphate buffer (0.1 M, pH 9), at room temperature for 5 min. The reaction was started by adding 20 µL of linolenic acid (4.18 mM in ethanol) and followed for 3 min at 234 nm. Results correspond to the mean ± SEM of three independent assays, each performed in triplicate. Quercetin was used as the positive control. For honey, 5 g was diluted in 10 mL of distilled water on the day of the essay and mixed well for 5 min using a vortex. The volume of solution used for the test was 150 µL [21].

### 2.6. Statistical Analysis

Results were expressed as mean ± standard deviation of triplicate analyses for all measurements. Analysis of variance (ANOVA) for comparison of sample means was used to analyze variations in observed parameters among the samples. All data were processed statistically using the software package SPSS.

## 3. Results and Discussion

### 3.1. Physicochemical Properties of Thymus Vulgaris Honey

The physicochemical properties of the studied thyme honey, including moisture, pH, free acidity, lactone acidity, electrical conductivity, sugar content, ash content, and hydroxymethylfurfural (HMF), are shown in Table 1.

Data showed that all the sample analytical parameters conformed to the limits within the Codex Alimentarius Standards. A great parameter to determine the quality of honey is moisture content, as it is directly associated with ecological factors, yielding season, maturity reached, honey storage, and the source of nectar used by the bees. The moisture content in the thyme honey sample (17.15 ± 0.30%) was within the international limit (≤21%), which suggests great quality, appropriate handling, and adequate storage of the honey. Honey is naturally acidic; this acidity may be due to the presence of organic acids that give it its flavor and allows it to resist microbial spoilage. The pH is also important because it affects the texture as well as stability and shelf life. Our sample showed a great acidic pH value (4.37 ± 0.26); the acidity can indicate the presence of many organic acids, such as gluconic acids and their lactones and esters. Acidity in honey varies due to floral origins and harvesting seasons. Both free acidity (35.88 ± 3.32 meq/Kg) and lactone acidity (3.33 ± 0.72 meq/Kg) of the thyme honey sample were within the international limits. The electrical conductivity value of the sample was 0.82 ± 0.083 ms/cm. This result suggests that the honey sample is from nectar honey, which is supported by the total ash content (0.27 ± 0.12%) of less than 0.6%. A great indicator of honey freshness is HMF since it is related to several factors, such as aging, storage conditions, pH, and concentration of metallic ions. Our sample showed a lower level than the maximum established by Codex Alimentarius Standards.

### 3.2. Chemical Composition Property of the Studied Essential Oils

The GC/MS analysis (Table 2) revealed that the major chemical compound detected in *E. globulus* EO was 1,8-cineole (90.14%). Similar results of the predominant compound (1,8-cineole) have been reported in the literature (55.9 and 55.29%) [57,58]. The main components for *O. vulgare* EO were thymol (54.21%) and carvacrol (19.08%), followed by benzene, 1,2,3,4-tetramethyl (8.15%), and γ-terpinene (8%). Carvacrol and thymol have been identified as major compounds of *O. vulgare* EO in other studies [37,59,60]. A total of 10 components have been identified in the EO of *M. spicata*, representing 88.98% of the total composition. The main components are carvone (60.37%), limonene (21.56%), cis-dihydro carvone (2.54%), 1,8-cineole (2.43%), and *β*-bourbonene (1.37%).

The anti-inflammatory potential of 1,8-cineole, the main component in *E. globulus* EO, was highlighted in numerous studies. Santos et al. studied the anti-inflammatory potential of 1,8-cineole using the colitis model induced by Tri-nitro-benzene-sulfonic acid (TNBS) in rats, which was one of the most common experimental models used to screen for drugs active against inflammatory disease. The treated rats with 1,8-cineole showed significantly reduced inflammatory damage scores [61]. The anti-inflammatory activity of *E. globulus* EO can be mediated by inhibiting the last phase by restricting the production of several cytokines, bradykinins, leukotrienes, and prostaglandins. Therefore, it quickly controlled both phases of inflammation [62]. Yoon et al. confirmed the relationship between the presence of 1,8-cineole as a selective COX-2 inhibitor and the significant inhibitory potential on PGE2 production [63]. Juergens et al. revealed that 1,8-cineole inhibited leukotrienes (LTB4) and prostaglandins (PGE2) [64].

The anti-inflammatory potential of the main components present in the EO of *O. vulgare* was brought to light in several studies, where carvacrol and thymol are explored as potent antimicrobial, antioxidant and anticancer compounds via inhibition of reactive oxygen species and free radicals production [65,66,67,68]. Terpinenes (e.g., γ- and α-terpinene) also showed excellent antioxidant, analgesic, anti-inflammatory, anti-proliferative, and antimicrobial effects [69]. Most importantly, terpinenes have been recently recognized as promising therapeutic agents suppressing microbial burden in skin injuries and dermatological disorders [70,71] via inhibition in the production of pro-inflammatory cytokines and other mediators in injured tissues [72].

The effectiveness of the anti-inflammatory potential of *M. spicata* EO was reported in several studies. Zhao et al. and Andrade et al. reported that the anti-inflammatory activities obtained with *M. spicata* EO might be related to some anti-inflammatory molecules, such as oxygenated monoterpenes (carvone and eucalyptol) [73,74]. In other studies, D-carvone inhibited the animal hind paw edema induced by various phlogistics (histamine, carrageenin, dextran, and bradykinin) in a dose-dependent manner [73,75].

Differences in the chemical composition of EOs can be found in other reports, which may relate to diverse abiotic or biotic factors, including extraction methods, climate and seasonal variations, geographical conditions, relative humidity, agronomic conditions (harvesting period, crop density, organic fertilizers), genotype, plant growth stage and treatment of plant materials prior to distillation [76,77,78].

Further studies are still required to explore and identify the definite underlying mechanisms that could be beyond the favorable therapeutic effect of some EOs in the anti-inflammatory process.

### 3.3. Antimicrobial Potential of the Studied EOs

The antibacterial assay was carried out to evaluate the possible prevention and disinfection power of the studied EOs against bacterial infection. EOs inhibition mechanisms involve multiple modes of action. It may be partly due to their hydrophobicity causing lipid partitioning of bacterial cell membranes, disturbing cell structures, and making them permeable [79,80]. It can also be explained by the lipophilic character of monoterpenes contained in EOs that can disrupt the microbial cytoplasmic membrane [81]. The exit of critical molecules and ions or extensive leakage from bacterial cells will lead to death [82].

Based on the disc-diffusion assay, as shown in Table 3, the three tested EOs showed significant antibacterial potential against tested bacteria compared to chloramphenicol (one-way ANOVA, *p* ≤ 0.05). However, some were more effective than others; *E. globulus* EO showed the highest inhibition zone, followed by *O. vulgare* EO, while the lowest inhibition zone was recorded by *M. spicata* EO.

The results obtained were in harmony with other published reports that claimed the potent antimicrobial effect of the essential oils of *O. vulgare* [83,84,85], *E. globulus* [86,87,88], and also *M. spicata* [89,90,91,92,93,94].

MIC and MBC are represented in Table 4. The results obtained support those of the disc-diffusion assay. The three EOs show low MIC and MBC values compared to the reference.

The most susceptible bacteria against *O. vulgare* EO were *S. aureus, L. monocytogenes, E. coli,* and *S. typhimurium,* respectively. For *E. globulus* EO, *L. monocytogenes* followed by *S. aureus* and *E. coli* and then *S. typhimurium*. In contrast, the microorganisms that were more sensitive to *M. spicata* EO were S. aureus, L. monocytogenes, E. coli, and S. typhimurium, respectively.

Indeed, the MIC test is required to evaluate the novel composite’s antibacterial properties traditionally used to treat inflammation [95]. To evaluate the nature of the antibacterial molecules, MBC/MIC values listed in (Table 4) have been calculated. All values were less than 4, meaning these essential oils have bactericidal effects on the examined bacteria. It was published that when the MBC/MIC ratio is 4, the extract is regarded as bactericidal, but when the MBC/MIC ratio is >4, it is deemed bacteriostatic [37,96]. Therefore, based on the antibacterial evaluation of the tested EOs, these components are strongly suggested for potential involvement in the inflammatory healing process.

### 3.4. Acute and Subacute Toxicity Studies of the Essential Oils

#### 3.4.1. Acute Toxicity

Acute toxicity is usually defined as the adverse change(s) occurring immediately or a short time following a single or short period of exposure to a substance or substances [97]. The objectives of this test are to identify the dose causing major adverse effects and also to estimate a minimum dose for the substance or material lethality [98]. Acute toxicity studies in animals are conducted using the same route of administration of the compounds intended for humans. Although LD_50_ does not predict a lethal dose in humans, it provides a guide to choosing a dose for subacute and sub-chronic studies. Daily administration of the lower dose in the toxicity study provides some indication of the long-term toxicity of the plant’s essential oils.

In our study and throughout the time of observation, there were no significant changes recorded in the behavior of any of the animals treated with *M. spicata* EO and no clinical signs of toxicity or treatment-related mortality during the observation period among the mice. The animals appeared very healthy, and their physical activity appeared normal. No abnormal changes attributable to treatment were noticed, such as respiration and heart rates. Therefore, the EO of *M. spicata* seems to be safe at a dose of 5000 mg/kg, i.p., and the LD_50_ is considered greater than 5000 mg/kg. Several studies have investigated the toxicity of the extracts of *M. spicata* [98,99,100] or that of other species [101]. But only two studies evaluated the acute toxicity of the EO of the plant [92,102]. The result of LD_50_ orally administered to rats was 5000 mg/kg, identical to our result.

*E. globulus* EO showed no effect on the mice at a dose of 1650 mg/kg and below, but caused signs of toxicity and death at a dose of 1750 mg/kg and above; the severity of these effects was increased within 6 h, and the mortality was 100%. The calculation LD50 gives the following results:

LD_50_ = 1795.7 mg/kg, 1771.8 < LD_50_ < 1819.94, with confidence limit at 95%. Similar results were found in other studies [103,104,105].

No toxic symptoms or death were observed among the mice treated with *O. vulgare* EO at all doses. The animals survived being active and healthy, and the LD_50_ is considered greater than 2000 mg/kg. Close results were found in the study by Selim et al., where the Acute toxicity test of *O. majorana* EO was performed on hamsters and showed complete survival and no occurred toxicity symptoms [106].

#### 3.4.2. Subacute Toxicity

The survival rate of the animals at the end of the experiment was 100%. We have not observed convulsions, numbness, or diarrhea nor detected alterations in the hair or skin. The color of the urine also remained normal. However, a decrease in body weight was observed in all groups treated with EOs (Table 5). Oppositely, the rats in the control group exhibited weight gain except on days 6 and 28, when slight decreases were spotted. The same results, except for body weight variation, were exposed by Llana et al. with a lack of toxic effects of *O. vulgare* EO given in the rats’ diet at doses of 50, 100, and 200 mg/kg b.w./d. Animals did not show any change in body weight, food, and water consumption or in biochemical and hematological parameters [107]. In line with the findings, Gebremickael et al. reported that acute and sub-chronic oral toxicity evaluation of *E. globulus* EO-water emulsion did not result in any toxicity-related mortalities and body weight change in mice [104]. Furthermore, no hazardous or adverse effects have been reported after the consumption of 0.24 mL of pure *M. spicata* EO daily for three continuous weeks in other studies [108,109].

Few toxicological investigations were found for the three EOs in the published scientific literature.

### 3.5. Anti-Inflammatory Activity

#### 3.5.1. In Vivo Anti-Inflammatory Tests

The inflammatory response of human tissue is usually associated with the presence of edema, particularly if the inflamed tissue targets the skin. The accumulation of fluid and white blood cells in the injured area is caused by the secretion of inflammatory cytokines into the bloodstream [3]. One of the methods extensively used to verify the anti-inflammatory activity of many drugs is the induction of paw edema by carrageenan [110]. After the carrageenan injection, edema occurs in two phases [111]; the first takes place between 0 and 150 min after the injection of carrageenan and has been attributed to serotonin, bradykinin, and histamine on vascular permeability. Serotonin and histamine are released for 1 hr 30 min, while bradykinin is released for 2 h 30 min after carrageenan injection [112,113,114]. The second phase occurs between 2 h 30 min and 6 h after the initiation of inflammation and contains the secretion of prostaglandins [112,115]. After six hours, the inhibition of carrageenan-induced inflammation stops [116].

**Figure 1:**
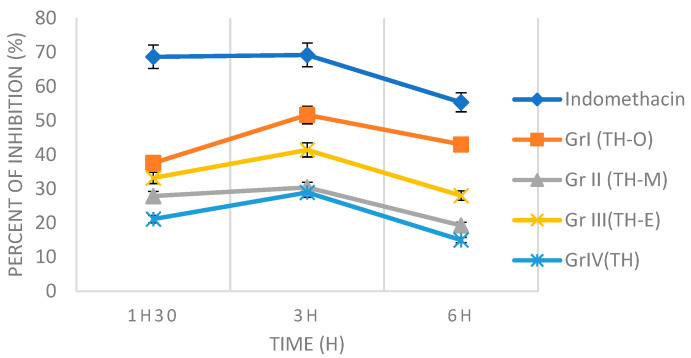
Effects of indomethacin, Thyme honey and mixtures on carrageenan-induced rat paw edema. TH: Thyme honey; TH-O: mixture of honey and *O. vulgare* EO; TH-M: mixture of honey and *M. spicata* EO; TH-E: mixture of honey and *E. globulus* EO.

**Figure 2:**
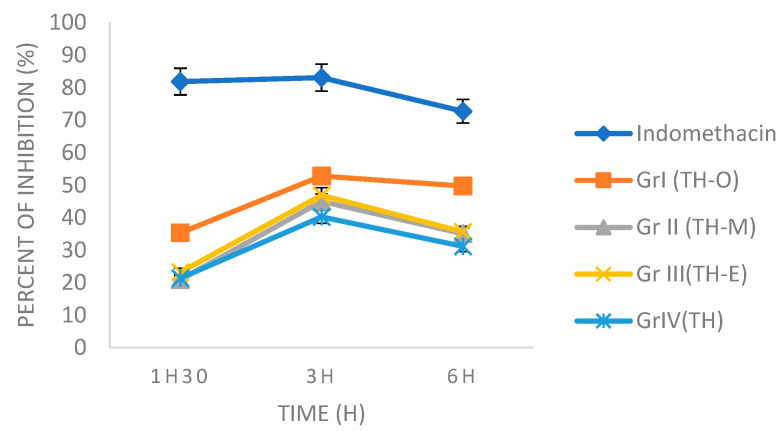
Effects of indomethacin, Thyme honey and mixtures on experimental trauma-induced rat paw edema. TH: Thyme honey; TH-O: mixture of honey and *O. vulgare* EO; TH-M: mixture of honey and *M. spicata* EO; TH-E: mixture of honey and *E. globulus* EO.

Table 6 and Table 7 show the effect of thyme honey and honey-essential oil mixtures on carrageenan-induced and trauma-induced inflammatory edema of the left rat paw. Subplantar injection of carrageenan to the control lot and experimental trauma resulted in a significant increase in the left paw injected with carrageenan, which peaked (0.546 ± 0.014 and 0.693 ± 0.016 mL, respectively) in paw volume 180 min after the induction. This confirms that both carrageenan injection and experimental trauma generated an acute inflammatory reaction in the left hind paw of the animal. Thyme honey and the three mixtures reduced the edema in the first and second phases of carrageenan inflammation (significance *p* < 0.05), and their effects on carrageenan edema were time-dependent (Table 8 and Figure 1). In the trauma-induced edema, honey and mixtures also showed great results and decreased edema in the different phases of inflammatory response (Table 9 and Figure 2).

Three hours after carrageenan administration, the mixture of thyme honey and *O. vulgare* EO showed the best inhibition activity with a peak effect of 51.65%, followed by the mixture of thyme honey and *E. globulus* EO (41.39%). Next were the mixtures of honey and *M. spicata* EO and thyme honey, with 30.41 and 28.94%, respectively (Table 8). The same results were observed in the experimental trauma-induced model with inhibition percentages (after 180 min) of 52.81% for the mixture of honey with the EO of *O. vulgaris*, followed by the mixture of honey with EO of *E. globulus* (46.90%), then the mixture of honey with EO of *M. spicata* with 45.02%, and lastly thyme honey with a percentage of inhibition of 40.26% (Table 9).

Several studies have reported suppression and relief symptoms of inflammation following the application or consumption of thyme honey. The study by Siphahi et al. showed that thyme honey inhibited the LPS-induced inflammation significantly and nearly neutralized it. LPS is a good stimulator of macrophages and leads them to produce more pro-inflammatory cytokines by the expression of nitric oxide synthase enzyme [117]. Inhibition of NO production in LPS-stimulated RAW 264.7 cells is one of the possible ways to screen various anti-inflammatory drugs [118,119]. Hashemian et al. revealed that thyme honey has an excellent safety profile for reducing inflammation [120]. Carvacrol, a compound found only in thyme honey [121], activates PPAR α and γ and suppresses COX-2 expression [122]. COX-2 enzyme plays an important role in inflammation [123]. Thus, it can be inferred that thyme honey can reduce inflammation.

Multiple studies highlighted the effectiveness of the three EOs used in our study in reducing inflammation, *O. vulgare*, *E. globulus,* and *M. spicata* EO showed anti-inflammatory properties through a significant reduction of ROS, ICAM-1, iNOS, COX-2, 8-OHdG, MMP-1, and MMP-12 [59,62,75,88,124]. Rare are the studies carried out on the combination of honey with EO, and to the best of our knowledge, none of them investigated anti-inflammatory activity.

In our study, the three mixtures showed great results regarding the inhibition of inflammation, even better than the honey alone. However, *O. vulgare* followed by *E. globulus* EOs best enhanced the anti-inflammatory potential of Thyme honey in both tests.

Although conclusive results have been found, the exact mechanism of synergy between essential oils and honey is unclear, and no data have been found in the literature. Further investigation should be done to isolate the bioactive molecules responsible for the anti-inflammatory potential and characterize the synergetic mechanism occurring between them.

**Figure 3:**
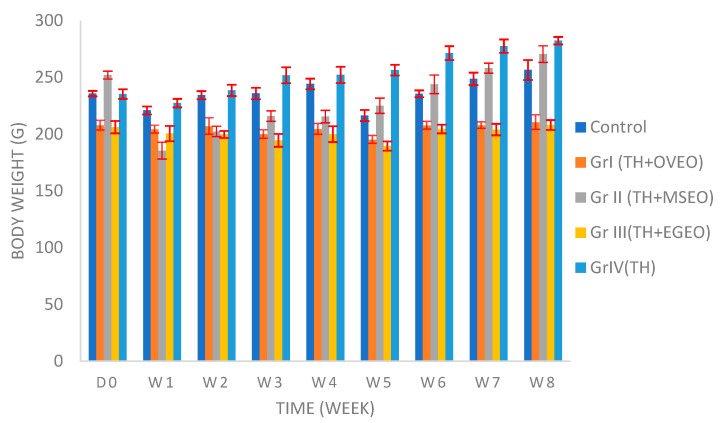
Representative graphs for body weight changes for rats treated with Thyme honey and mixtures of honey with essential oils. TH: thyme honey, OVEO: *O. vulgare* essential oil, MSEO: *M. spicata* essential oil, EGEO: *E. globulus* essential oils.

Figure 3 shows the effect of honey and mixtures on the body weight of rats during two months of treatment. A decrease in body weight in all groups was observed in the first week of treatment, which may be due to the change in the diet of animals or stress factors. In the following weeks, a constant increase in body weight was spotted in GrII treated with a mixture of honey and *M. spicata* EO and GrIV receiving thyme honey. In contrast, the body weight of GrI and GrIII kept increasing and decreasing in weeks 2 to 5 before increasing slightly again in weeks 6, 7, and 8. Administration of mixtures of honey and EOs for two months significantly prevented overall weight gain in rats compared to the control group or the rats receiving only honey.

The anti-obesity effect of honey was further demonstrated in multiple studies compared to a sugar-free diet or sucrose and mixed sugar diets [125,126,127].

Several studies also investigated EOs as potential dietary supplements for weight loss. Earlier research has shown that the flavor of the citron group from grapefruit (*Citrus paradisi*) and lemon (*Citrus limonia*) essential oil increased the sympathetic nerve activity to white adipose tissue in anesthetized rats, suggesting increased lipolysis and suppressed body weight gain [128]; sweet orange EO reduced body weight gain and fat rate in obese rats [129], and administration of lime (*Citrus aurantifolia*) essential oil to mice caused significant suppression in gaining weight [130]. *Pinus koraiensis* EO markedly blocked obesity via the inhibition of lipid metabolism, including that of triglycerides [131]. Thus, based on the literature, EOs from the citrus family are the most investigated in obesity studies, and only a few were found regarding the essential oils in our study. Oregano EO was designated as an efficient dietary supplement to alleviate transport stress in finishing pigs [132]. Spearmint (*Mentha spicata*) EO reduced body weight in rats [133], while *Eucalyptus globulus* EO at a dose of 250 mg/kg has led to weight gain in broiler chickens [134]. The anti-obesity effects of EOs may be mediated via several mechanisms, including anti-lipase activity, anti-hyperlipidemia, downregulation of adipogenic transcription factors, and suppression of fat accumulation and intracellular triglycerides [135].

No results were found regarding weight gain/loss from honey mixtures with essential oils. Therefore, more studies should be conducted to evaluate their synergy.

#### 3.5.2. In Vitro Anti-Inflammatory Test (5-Lipoxygenase (5-LOX) Inhibition Ass)

Thyme honey and EOs were evaluated for their in vitro anti-inflammatory activity compared to the standard quercetin, and the results are shown in Table 10. Thyme honey inhibited the enzyme with IC_50_ = 29.53± 0.17 µg/mL, EOs of *O. vulgare*, *E. globulus,* and *M. spicata* with IC_50_ values of 13.23 ± 0.02, 15.53 ± 0.17, and 27.14 ± 0.07 (µg/mL), respectively, while IC_50_ = 1.09 ± 0.05 µg/mL was observed for quercetin under the same conditions. Thyme honey and *M. spicata* EO exhibited good anti-inflammatory. However, the essential oils of *O. vulgare* and *E. globulus* exhibited high anti-inflammatory activity.

LOX enzyme inhibition activity data agree with the results of the anti-inflammatory activity in vivo and showed a similar structure–activity relationship pattern. It has been stated that good inhibitory action on 5-LOX contributes to anti-inflammatory activity [136].

## 4. Conclusions

In this work, the anti-inflammatory activity of Moroccan thyme was evaluated and compared with the mixtures between the same honey and three essential oils to enhance the synergy between these two natural components. The tests were performed *in-vivo* on two models of inflammation in rats. For both models, the mixtures were more effective in reducing inflammation volume than honey alone. However, the mixtures that provided the best results were those of honey with *O. vulgare* EO followed by the mixture of honey with *E. globulus* EO. The two mixtures may be used for their great anti-inflammatory potential in humans after certain clinical trials.

## Figures and Tables

**Table 1:** Physicochemical characteristics of thyme honey sample.

Sample	Moisture (%)	pH	Free Acidity (meq kg^−1^)	Lactonic Acidity (meq kg^−1^)	Total Acid-ity (meq kg^−1^)	Electrical Conductivity (ms cm^−1^)	Sugar Conten (°Brix)	Ash (%)	HMF (mg/kg)
THYME HONEY	17.15 ± 0.30	4.37 ± 0.26	35.88 ± 3.32	3.33 ± 0.72	39.21 ± 2.72	0.82 ± 0.083	79.83 ± 2.46	0.27 ± 0.12	8.44 ± 3.43
CODEX	≤21%	3.4–6.1	≤50 meq/kg	_	8.68–59.49 meq/kg	≥0.700 (ms cm^−1^)	≥60 °Brix	≤0.6%	≤40 mg/kg

Note: Values are expressed as mean ± SD (n = 3). HMF: Hydroxymethylfurfural.

**Table 2:** Chemical composition of the studied EOs.

NO	*O. vulgare* EO ^a^			*M. spicata* EO ^b^			*E. globulus* EO ^c^		
	Compound	RT *	%	Compound	RT	%	Compound	RT	%
1	α-Pinene	1.390	0.4	α-Pinene	1.356	0.84	α-Pinene	2.093	3.85
2	Camphene	1.502	0.09	Camphene	1.457	0.24	Camphene	2.251	0.48
3	β-Phellandrene	2.257	0.78	β-Pinene	1.762	1.84	2,4-Thujadiene	2.363	0.09
4	2-Carene	2.483	1.04	Limonene	2.697	21.56	β-Pinene	2.713	0.62
5	Benzene, 1,2,3,4-tetramethyl	2.764	8.15	3-Octanol, acetate	5.154	0.43	β-Myrcene	3.186	0.58
6	p-Cymene	3.114	0.68	Borneol	5.638	1.00	α-Phellandrene	3.299	0.96
7	γ-Terpinene	3.441	8.00	Terpinen-4-ol	5.931	0.4	Eucalyptol (1,8-cineol)	4.268	90.14
8	Benzene, 2-butenyl	4.579	0.12	Carvone	8.050	60.37	β-Ocimene	4.380	0.28
9	Linalool	5.029	1.01	β-Bourbonene	10.596	2.30	*γ*-Terpinene	4.651	2.39
10	α-Terpineol	6.280	0.24	Caryophyllene	11.385	1.51	p-Cymenene	5.237	0.08
11	Thymol	10.247	54.21	__	__	__	2,4,6-Octatriene, 3,4-dimethyl	6.330	0.07
12	Carvacrol	10.867	19.08	__	__	__	trans-Sabinol	7.952	0.08
13	Caryophyllene	11.757	2.26	__	__	__	1_7_7-Trimethylbicyclo_2.2.1_hept-5-en-2-ol	10.330	0.09
14	Humulene	12.703	0.11	__	__	__	__	__	__
15	σ-Cadinene	15.149	0.20	__	__	__	__	__	__
Total identified compounds%		96.57		88.98			99.71		
Monoterpene hydrocarbons%		19.26		24.48			9.4		
Oxygenated monoterpenes %		74.54		62.2			90.31		
Sesquiterpenes hydrocarbons%		2.57		2.3			__		
Oxygenated sesquiterpenes%		__		__			__		

**^a^***Origanum vulgare* essential oil; **^b^** *Mentha spicata* essential oil; **^c^** *Eucalyptus globulus* essential oil. * RT = Retention time.

**Table 3:** The inhibitory diameters (mm) of EOs of *O. vulgare, E. globulus,* and *M. spicata* compared to chloramphenicol (30 μg/disc).

Microorganisms	Mean Zone of Inhibition in Millimeters (Mean ± Standard Deviation) *			
	*O. vulgare* EO	*E. globulus* EO	*M. spicata* EO	Chloramphenicol (30 μg)
*E. coli* ATCC 25922	25.1 ± 0.4	28.3 ± 1.1	21.5 ± 0.5	21.0 ± 0.4
*S. typhimurium* ATCC 700408	23.1 ± 0.6	21.8 ± 0.9	17.5 ± 0.6	12.6 ± 0.4
*S. aureus* ATCC 29213	30.4 ± 0.9	32 ± 1.43	26.8 ± 1.1	23.0 ± 0.6
*L. monocytogenes* ATCC 13932	34.4 ± 1.2	36 ± 0.5	22.6 ± 0.9	26.7 ± 0.9

Diameters in mm, * Mean of three replicates.

**Table 4:** MIC and MBC values of the essential oils of *O. vulgare, E. globulus* and *M. spicata* *.

Microorganisms	*O. vulgare* EO		*E. globulus* EO		*M. spicata* EO		Chloramphenicol	
	MIC (mg/mL)	MBC (mg/mL)	MIC (mg/mL)	MBC (mg/mL)	MIC (mg/mL)	MBC (mg/mL)	MIC (μg/mL)	MBC (μg/mL)
*E. coli* ATCC 25922	1.56 ± 0.00	1.56 ± 0.11	1.56 ± 0.26	1.56 ± 0.03	1.68 ± 0.024	3.12 ± 0.093	4	4
*S. typhimurium* ATCC 700408	3.12 ± 0.221	3.12 ± 0.72	3.12 ± 0.83	4.5 ± 1.44	12.5 ± 0.09	25 ± 0.219	64	64
*S. aureus* ATCC 29213	0.78 ± 0.11	0.78 ± 0.20	0.78 ± 0.09	0.78 ± 0.00	1.56 ± 0.36	2.56 ± 0.008	4	4
*L. monocytogenes* ATCC 13932	0.78 ± 0.018	0.78 ± 0.00	0.68 ± 0.36	1.56 ± 0.00	1.56 ± 0.018	3.12 ± 0.048	2	2

* MIC and MBC values of EOs are interpreted in mg/mL, and the standard antibiotic is interpreted in μg/mL.

**Table 5:** Body weight (g) of rats treated with essential oils of *M. spicata*, *E. globulus,* and *O. vulgare* for 28 days and percentage of weight gain (%).

Groups	Treatment	Daily Dose (mg/Kg)	Weight & Weight Gain Percentage	Day 1	Day 6	Day 10	Day 14	Day 18	Day 22	Day 28
Gr I	*M. spicata* EO	5000	Weight (g) SD Weight gain (%)	245.66 ± 3.61 —	242.36 ±3.76 −1.34	244.94 ±4.11 −0.29	238.3 ±4.84 −3.00	237.98 ±4.17 −3.12	240.5 ±3.97 −2.10	244.26 ±3.71. −0.57
Gr II	*E. globulus* EO	1759.7	Weight (g) SD Weight gain (%)	256.72 ±2.80 —	245.3 ±1.87 −4.45	242.08 ±3.14 −5.70	243.97 ±2.77 −4.97	242.6 ±2.41 −5.50	238.93 ±3.62 −6.93	249.32 ±3.44 −2.88
Gr III	*O. vulgare* EO	2000	Weight (g) SD Weight gain (%)	232.96 ±1.46 —	227.68 ±2.21 −2.66	224.56 ±2.72 −3.60	223.32 ±3.94 −4.14	221.48 ±3.72 −4.92	228.15 ±4.68 −1.80	223.02 ±5.01 −4.27
Control	—	—	Weight (g) SD Weight gain (%)	248.76 ±4.73 —	246.68 ±3.19 −0.84	259.68 ±3.62 4.39	264.54 ±3.73 6.34	256.71 ±4.11 3.20	253.9 ±2.64 2.07	247.43 ±3.42 −0.53

SD: standard deviation (n = 6).

**Table 6:** Effect of thyme honey and mixtures on carrageenan-induced rat paw edema.

Treatment Group	Mean Edema Volume (Left-Right Paw) mL		
	1 h 30 min	3 h	6 h
Control	0.431 ± 0.011	0.546 ± 0.014	0.421 ± 0.02
Indomethacin (10 mg/Kg)	0.135 ± 0.014 *	0.168 ± 0.016 *	0.188 ± 0.017 *
GrI (mixture of TH and *O. vulgare* EO)	0.269 ± 0.015 *	0.264 ± 0.014 *	0.240 ± 0.011 *
Gr II (mixture of TH and *M. spicata* EO)	0.311 ± 0.017 *	0.381 ± 0.018 *	0.341 ± 0.013 *
Gr III (mixture of TH and *E. globulus* EO)	0.288 ± 0.014 *	0.320 ± 0.013 *	0.280 ± 0.014 *
GrIV (TH)	0.340 ± 0.016 *	0.388 ± 0.014 *	0.358 ± 0.019 *

Note: Values are expressed as mean ± SD; SD, standard deviation; (n = 6 of each group). Abbreviation: n, number of rats. * *p* < 0.05 statically significant compared to the control. The reference drug is indomethacin. TH: thyme honey.

**Table 7:** Effect of thyme honey and mixtures on trauma-induced rat paw edema.

Treatment Group	Mean Edema Volume (Left-Right Paw) mL		
	1 h 30 min	3 h	6 h
Control	0.43 ± 0.014	0.693 ± 0.016	0.539 ± 0.014
Indomethacin (20 mg/Kg)	0.078 ± 0.013 *	0.117 ± 0.019 *	0.147 ± 0.014 *
GrI (mixture of TH and *O. vulgare* EO)	0.278 ± 0.014 *	0.327 ± 0.017 *	0.271 ± 0.016 *
Gr II (mixture of TH and *M. spicata* EO)	0.340 ± 0.013 *	0.382 ± 0.014 *	0.351 ± 0.017 *
Gr III (mixture of TH and *E. globulus* EO)	0.331± 0.014 *	0.368 ± 0.016 *	0.347 ± 0.019 *
GrIV (TH)	0.338 ± 0.016 *	0.414 ± 0.013 *	0.371 ± 0.014 *

Note: Values are expressed as mean ± SD; SD, standards deviation; (n = 6 of each group). Abbreviation: n, number of rats. * p < 0.05 statically significant compared to the control. The reference drug is indomethacin. TH: thyme honey.

**Table 8:** Percentage inflammation inhibition by thyme honey and mixtures on carrageenan-induced rat paw edema.

Treatment Group	Percentage Inhibition of Edema (%)		
	1 h 30 min	3 h	6 h
Indomethacin (10 mg/Kg)	68.67	69.23	55.34
GrI (mixture of TH and *O. vulgare* EO)	37.58 *	51.65 *	42.99 *
Gr II (mixture of TH and *M. spicata* EO)	27.84 *	30.41 *	19.24 *
Gr III (mixture of TH and *E. globulus* EO)	33.18 *	41.39 *	28.01 *
GrIV(TH)	21.11 *	28.94 *	14.96 *

Notes: * *p* < 0.05 statically significant compared to the reference drug (indomethacin). TH: thyme honey.

**Table 9:** Percentage inflammation inhibition by Thyme honey and mixtures on experimental trauma-induced rat paw edema.

Treatment Group	Percentage Inhibition of Edema (%)		
	1 h 30 min	3 h	6 h
Indomethacin (20 mg/Kg)	81.86	83.12	72.71
GrI (mixture of TH and *O. vulgare* EO)	35.45 *	52.81 *	49.72 *
Gr II (mixture of TH and *M. spicata* EO)	20.93 *	45.02 *	35.06 *
Gr III (mixture of TH and *E. globulus* EO)	23.25 *	46.90 *	35.62 *
GrIV (TH)	21.39 *	40.26 *	31.17 *

Notes: * *p* < 0.05 statically significant compared to the reference drug (indomethacin). TH: thyme honey

**Table 10:** Anti-inflammatory activity of Thyme honey and EOs of *M. spicata*, *O; vulgare,* and *E. globulus*.

	TH	*M. spicata* EO	*O. vulgare* EO	*E. globulus* EO	Quercetin
IC_50_ (µg/mL)	29.53 ± 0.17 ^d^	37.14 ± 0.07 ^e^	13.23 ± 0.02 ^b^	15.53 ± 0.17 ^c^	1.09 ± 0.05 ^a^

Different letters indicate significant differences (*p* < 0.05; n = 3). TH: thyme honey, EO: essential oil. IC_50_: Half-maximal inhibitory concentration.

## Data Availability

The data used to support the findings of this study are included within the article.
